# Prevalence and Related Risk Factors of Intestinal Parasitosis among Private School-Going Pupils of Dharan Submetropolitan City, Nepal

**DOI:** 10.1155/2021/6632469

**Published:** 2021-07-09

**Authors:** Bijay Kumar Shrestha, Manita Tumbahangphe, Jenish Shakya, Anu Rai, Kabita Dhakal, Bidhya Dhungana, Romika Shrestha, Jyoti Limbu, Kabiraj Khadka, Santoshi Ghimire, Sujata Chauhan, Lata Chalise, Ashu Ghimire

**Affiliations:** Department of Microbiology, Central Campus of Technology, Tribhuvan University, Hattisar, Dharan, Nepal

## Abstract

**Introduction:**

Intestinal parasitic infections are most common and prevalent among children and accounts for great morbidity and mortality.

**Objective:**

This research is aimed at studying the prevalence and related risk factors of parasitic infections among private school-going pupils of Dharan Submetropolitan City.

**Methods and Materials:**

This was a cross-sectional laboratory-based study conducted from 13 November 2018 to 26 February 2019 among 400 private school pupils. The stool samples were collected and microscopically examined for parasites using the formalin ethyl acetate sedimentation technique. *Data Analysis*. Statistical analysis was performed by using SPSS version 16.0. Pearson's Chi-square test was used to establish association between dependent and independent variables. The association was also determined using crude and adjusted odds ratio, and the test considered a *P* value < 0.05 as statistically significant with 95% confidence interval.

**Result:**

In this study, 46 (11.5%) children were positive for intestinal parasites. In this study, 3 protozoans (*Entamoeba histolytica* = 3 (0.75%)) and 43 helminths (*Ascaris lumbricoides* = 22 (5.5%); *Enterobiusvermicularis* = 6 (1.5%); *Ancylostoma duodenale* = 2 (0.5%); and *Trichuris trichiura* = 13 (3.25%)) were isolated and identified. Statistically, significant difference in the parasitic prevalence with respect to age and gender was not seen (*P* > 0.05). However, the prevalence of parasitic infection was strongly associated with the ethnicity of the pupils (*P* = 0.001). The strong associated risk factors of intestinal parasitic infections were nail-biting habit, source of drinking water, biannual deworming, thumb-sucking, hand sanitation before having food and after toilet, knowledge of parents on parasitosis, health and sanitation, keeping cat/dog as pet, and wearing protective shoes during play (*P* = 0.001). Bowel syndromes like abdominal cramp and constipation also had a strong statistical association (*P* = 0.001) with the prevalence of parasitic infection. According to binary and multivariate logistic regression analyses, the parents without awareness, pupils with a nail-biting habit, pupils not wearing shoes during play, lack of deworming, drinking direct tap water, and pupils with poor hand sanitation were more likely to be infested with intestinal parasitic infections.

**Conclusion:**

This study concludes that intestinal parasites are still prevalent among private school-going pupils of Dharan Submetropolitan City. The poor sanitation and sanitary habits like biting nails, consumption of untreated drinking water, and failure to practice proper hand washing were studied as contributors to the acquisition of intestinal parasitic infections. Therefore, integration of control measures such as provision of clean and safe drinking water, improved sanitation and hygiene, with biannual administration of drugs are necessary for effective eradication of parasitic infections.

## 1. Introduction

Parasites are organisms that live and obtain their food from the host and are classified as protozoa, helminths, and ectoparasites [1]. The geographical distribution of the parasitic infection is influenced by various factors including environmental conditions like soil; lack of safe water supply; absence of sanitary facilities; unsafe waste disposal system; types of toilet; and human factors like age, sex, socioeconomic status, and occupation [2].

According to different researches conducted in recent years, the situation of parasitic infection in Nepal is deplorable. The rate of morbidity and mortality linked with parasitic infection is high in Nepal [3]. People of all age groups and gender are affected by parasitosis [4]. However, children, females, and youths (21 to 40 years) are more prone to the intestinal parasitic infection in Nepal [5, 6]. In fact, intestinal parasitosis has been found to be responsible for malnutrition and anemia with a high death rate in pregnant women and children [7]. Open defecation in rural areas of Nepal is a major reason behind the higher prevalence of parasitosis. Over population, poor hygiene, polluted drinking water, poor sanitation, illiteracy, lack of awareness, unhealthy food, farming occupation, socioeconomic condition, and cultural practices are considered to be important lagging factors responsible for the increasing rate of parasitic diseases in Nepal [6, 8–10]. *Ascaris lumbricoides*, *Hymenolepis nana*, and *Trichuris trichiura* are the most common helminths, while *Giardia lamblia* and *Entamoeba histolytica* are the most common protozoans causing parasitosis in Nepal [11]. Nevertheless, *Ascaris* is the most dominant helmint, and *Entamoeba* is the most prevalent protozoan among the underprivileged communities of Nepal [12]. The prevalence rate of parasitic infection in Nepal is different in different studies ranging from nearly 20% to even 60% in the overcrowded and polluted city of Nepal [9, 13].

School-aged children are at high-risk of intestinal parasitic infection which may have an impact on their mental and physical growth [14, 15]. Some behavioral aberrations such as finger sucking, encopresis, and nail biting observed in some children have been postulated as important risk factors that may encourage soil contamination by helminths and intestinal parasitic transmission from one individual to another [16].

Globally, 1.5 billion people are infected with soil-transmitted helminths, and above 267 million preschool children and over 568 million school-aged pupils live in intestinal helminth-prevalent areas [17]. Among the intestinal parasites, *Entamoeba histolytica* causes the death of more than 100,000 people annually. Similarly, *Giardia lamblia* affects approximately 200 million people worldwide which is followed by *Ascaris lumbricoides* and *Trichuris trichiura* affecting 1.4 billion and 1 billion people, respectively [18]. In Ethiopia, parasitic infections are the second most predominant cause of outpatient morbidity [19].

In Nepal, parasites account for 50% of diarrheal diseases among children with diarrhea recognized as the major killer of Nepalese children [20]. The age group of 5 years and below are considered a high-risk population to parasitic infection as compared to the higher-age groups which concords with findings of other studies in Nepal [20, 21]. In the worst scenarios, parasitic infection may impact health as well as bring about malnutrition leading to mental and physical growth retardation of children. In addition, it leads to iron deficiency, anemia, malabsorption syndrome, and intestinal obstruction [15, 22].

The government of Nepal has conducted several deworming programs in collaboration with World Health Organization (WHO) and other governmental and nongovernmental organizations. It is estimated that more than 20 million tablets of albendazole (an anthelminthic drug) have been distributed by female health workers to young school-going children of Nepal [23]. A biannual deworming program was initiated by the government of Nepal in the year 2000 A.D. in few districts of Nepal with an objective of providing antiparasitic drugs to the children-aged group (12-59 months) twice a year. The project added supplementation of vitamin A capsules to children with the aim of reducing the cases of anemia and load of parasites. This pilot project was then implemented all over the country by the end of 2010 A.D. because of its positive outcome [24]. Similarly, in the year 2006 A.D., the government of Nepal launched another biannual deworming project targeting children (class 1 to 5) going to public schools in 24 districts of Nepal. The high acceptance rate of this program made it expand further to 43 districts in 2009 A.D. [25]. At present, this project is in practice all over Nepal focusing on children (classes 1 to 10) of both public and private schools, but the reports of the impact of this program are not up to date [26]. In addition, the government of Nepal has recently released a program (2018-2022 A.D.) in cooperation with United Nations International Children's Emergency Fund (UNICEF) with the major goal of providing proper nutrition to infants, young children, pregnant mothers, and breast-feeding mothers by running deworming programs [27]. In developing countries like Nepal, intestinal parasitic infection is one the major causes of public health problems [25]. In Dharan, only few small-scale studies estimating the prevalence and/or assessing the associated risk factors of intestinal parasitic infections among pupils have been conducted. Therefore, this study was designed to address the information gap pertaining to the prevalence and associated risk factors of intestinal parasitosis. Therefore, this research was aimed at studying the prevalence and associated risk factors of intestinal parasitosis among private school-going pupils of Dharan Submetropolitan City.

## 2. Methods and Materials

### 2.1. Study Design and Study Population

This was a cross-sectional laboratory-based study conducted from 13 November 2018 to 26 February 2019 A.D. among four private school-going pupils of Dharan Submetropolitan City.

### 2.2. Study Area

**Figure 1:**
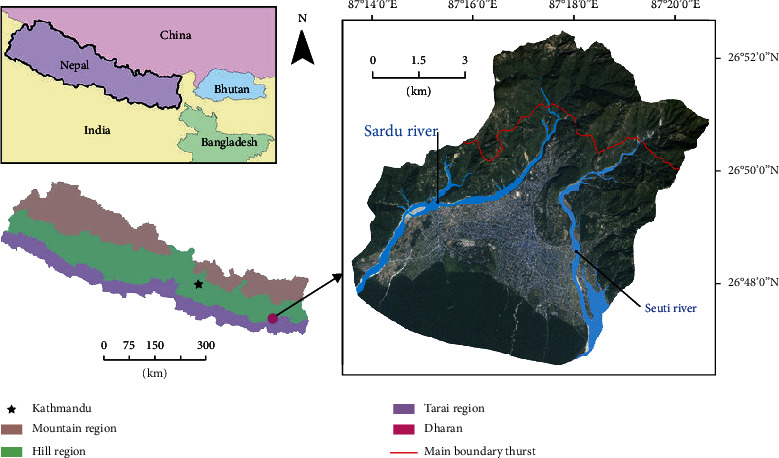
Map of Nepal representing the study site: Dharan Submetropolitan City (obtained from Dharan Submetropolitan City Office, Nepal).

Dharan is a city of the Sunsari District, Province no. 1 (latitude 26°49′12^″^N and longitude 87°18′0^″^E), Nepal, situated at an altitude of 349 m. Dharan is a small submetropolitan city in Nepal located at the base of hills (Mahabharat range) in the northern side and terai region in the southern part with a total area of 192.32 km^2^ (Figure 1). It is surrounded by Seuti River in the east and Sardu River in the west with a tropical monsoon climate. The warm temperate climate and monsoon rainfall of Dharan can be considered to be one of the factors responsible for transmission of parasitosis in Dharan [28]. According to the WHO report, the occurrence of intestinal parasitosis is higher during the period of the tropical rainy season, since the land becomes wet and moist which facilitates easy contact with parasitic larvae. Shortage of clean drinking water, poor hygiene among children like not using soap for washing hands after defecation, lack of knowledge about anthelminthic drugs, and lack of education are important limiting features of Dharan for easy spread of parasitic diseases [29]. Lack of awareness among parents about transmission of parasitosis was also found to be one of the reasons behind the persisting prevalence of parasitosis in Dharan. Furthermore, open defecation, lack of water filtration, lack of chlorinating techniques for drinking water, improper hand washing, poor financial condition, farming occupation, lower maintenance of personal hygiene, and use of pit latrines located near water sources which may contaminate the river are the remaining hallmarks of Dharan contributing to the transmission of intestinal parasitic infection [30].

### 2.3. Sample Size and Population

The sample size was calculated as 400 based on the parasite's prevalence of 22.5% [30], 95% confidence level, and 5% allowable error using the sample size prevalence formula, *n* = *z*^2^*pq*/*e*^2^, where *z* = 1.96, *p* = 0.225, *q* = 0.775, and *e* = 0.05. For this study, approval was obtained from the ethical review board and Department of Microbiology, Central Campus of Technology, Tribhuvan University, Dharan. Approval was taken from all the four private schools under study. Private school-going children under the age of 12 was set as a sampling frame. A sample unit was selected equally from all four private schools with 100 children from each private school. Private schools were selected for this study although similar studies were done before, but the data are too old and private schools were most often excluded in studies with an assumption that the good hygienic profiles maintained by pupils of private schools rule out the risk of parasitic infections. Since, in recent years the children from underprivileged community are also more often found in private schools. Therefore, it was foremost to screen the private schools for unnoticed parasitic infestation among pupils. Informed consent was obtained from parents on behalf of their children in a written form. Simple random sampling with a lottery method was used to select the private schools and pupils. Pupils under the age of 12 from nursery class to Grade V and who were present during the visit, interested, and allowed to participate were included in the study. A pupil's sociodemographic information like age, sex, and ethnicity; sanitary habits like nail-biting habit; source of drinking water; biannual deworming; thumb sucking; hand sanitation before having food and after using toilets; knowledge of parents on parasitosis, health, and sanitation; keeping cat/dog as pet; wearing protective shoes; and bowel syndrome were obtained through a semistructured questionnaire filled by the parents.

### 2.4. Sample Collection and Transportation

Parents were provided with a well-labeled, dry, clean, disinfectant-free wide-mouth plastic container with 5 mL of 10% formalin and were instructed to collect about 5 grams of stool from their children. Parents were instructed to collect stool till a reading level mark of the container which can rack up about 5 grams of stool. The containers were well labeled with children's code number, name, date, and time of collection. The sample without any label and which was contaminated with water, mud, and urine was excluded from the study. The stool samples collected by parents the next day was stored in an ice cool box and transported to the Microbiology Laboratory of the Central Campus of Technology, Dharan, and was examined within 2 hours.

### 2.5. Macroscopic and Microscopic Examination

The stool samples were macroscopically examined for the color, consistency, mucus, blood, and helminths. The stool samples were immediately fixed in an equal volume of 10% formalin. Microscopic observation of the stool sample was performed by the formalin ethyl acetate sedimentation technique as described by CDC [31]. For the sedimentation method, formalin-fixed stool samples were mixed well and strained through a cotton gauze into a 25 mL centrifuge tube. About 10 mL of 10% formalin was added through gauze on debris. The sample was centrifuged at 500 g for 10 minutes. The supernatant was discarded, and 10 mL of 10% formalin was added to the sediment and was mixed well by the use of a wooden applicator. About 4 mL of ethyl acetate was added and shaken vigorously in an inverted position for 30 seconds. The tube was centrifuged at 500 g for 10 minutes, and the supernatant was discarded. A cotton-tipped applicator was used to remove debris, and a few drops of formalin was suspended to a concentrated specimen. The specimen was mounted on a clean, grease-free glass slide, and the cover slip was placed over it and examined. A microscope was first set at 10x power lens then to high-power oil immersion of 100x lens to observe ova and cyst of parasites. The sociodemographic data and risk factors associated with parasitic infestation collected through the questionnaire were documented and examined for statistical analysis.

## 3. Data Analysis

The statistical analysis was performed using SPSS version 16.0. Frequency, percentages, and tables were used to present the results. Prevalence was calculated by measuring the presence of parasites in a sample of the population selected randomly, then dividing by the number of children of the sample population and multiplied by 100 to express the result of prevalence in percentage (%). Pearson's Chi-square test was used to establish association between dependent and independent variables. The association between variables was also determined by using odds ratio. Logistic regression was used to detect risk factors of parasite infection. The test was considered statistically significant at *P* value < 0.05 with 95% confidence interval.

## 4. Quality Control

Three days of training were provided to the researchers and supervisors before the study. One day of training was given to the parents for filling the questionnaire and were instructed on the procedure of sample collection. The questionnaire was prepared in Nepali language. Before data collection, 10% of pretest was done at the schools. The quality of collected data was checked by the principal investigator and supervisors daily. Cross-checking and proofreading were performed by the principal investigator to maintain the quality of the data.

## 5. Result

### 5.1. Prevalence of Parasites

Of the total 400 school children included in the study, 46 (11.5%) were positive for intestinal parasites. In this study, *Entamoeba histolytica* was present in three (3) participants with a prevalence of 0.75%, along with 43 helminths (*Ascaris lumbricoides*, *Entamoeba histolytica*, *Ancylostoma duodenale*, *Trichuris trichiura*). Among the helminths, the most prevalent was *Ascaris lumbricoides* with 22 (5.5%), followed by *Trichuris trichiura* with 13 (3.25%), *Enterobiusvermicularis* with 6 (1.5%), and *Ancylostoma duodenale* with 2 (0.5%) (Table 1).

### 5.2. Gender-Wise Distribution of Parasitic Infection

Among 46 positive stool samples, parasitic infection was high among male (27; 14.28%) than among female (19; 9%) pupils. Gender was not significantly associated with parasitic infection (*P* = 0.098) (Table 2).

### 5.3. Age-Wise Distributions of Parasitic Infection

In this study, out of 46 positive samples, children of the 6–8-year age group had the highest prevalence of parasitic infection (32; 13.55%), followed by the 9–11-year age group (14; 8.53%). There was no significant difference between age group and parasitic infection (*P* = 0.121) (Table 3).

### 5.4. Ethnicity-Wise Distribution of Parasitic Infection

In this study, intestinal parasitic infection was high among the Janajati (16; 11.76%), followed by the Madhesi (11; 23.91%) and the Dalit (10; 47.61%). The least prevalence was reported among the Brahmin (4; 3.38%) and the Chettri (5; 5.43%). There was significant difference between ethnicity and parasitic infection (*P* = 0.001) (Table 4).

### 5.5. Risk Factors Associated with Infestation of Intestinal Parasitic Infections

Among the positive 46 samples, the prevalence of intestinal parasites was found more in pupils with a thumb-sucking habit (35; 48.61%) than in pupils with no thumb-sucking habit (11; 3.35%). There was significant difference between thumb-sucking habit and parasitic infection (*P* = 0.001). The prevalence of intestinal parasites was found more in pupils whose parents were unaware of parasitosis and sanitation (36; 23.37%) than in pupils whose parents were aware of parasitosis and sanitation (10; 4.06%). There was significant difference between parent's awareness and parasitic infection (*P* = 0.001). The prevalence of intestinal parasites was found more in pupils with a nail-biting habit (35; 19.02%) than in children without a nail-biting habit (11; 5.09%). There was significant difference between the nail-biting habit and parasitosis (*P* = 0.001). The prevalence of intestinal parasites was found more in pupils without the habit of wearing protective shoes (32; 56.14%) than in pupils with the habit of wearing protective shoes (14; 4.08%). There was significant difference between the habit of wearing protective shoes and parasitosis (*P* = 0.001). The prevalence of intestinal parasites was found more in pupils with a cat/dog kept as a pet (34; 18.37%) than in pupils without pet animals (12; 5.58%). There was significant difference between pet-keeping status and parasitosis (*P* = 0.001). The prevalence of intestinal parasites was found more in pupils who missed biannual deworming (43; 81.13%) than in pupils who participated in deworming (3; 0.86%). There was a significant difference between deworming status and parasitosis (*P* = 0.001). The prevalence of intestinal parasites was found more in pupils drinking direct tap water (39; 16.95%) than in pupils drinking filtered/boiled water (7; 4.11%). There was significant difference between source of drinking water and parasitic infection (*P* = 0.001). The parasitic infection was found higher in pupils who do not wash their hands with soap and water (28; 38.88%) than in those who wash their hands with soap and water (18; 5.48%). There was significant difference between hand-wash hygiene (before meal and after use of latrine) and prevalence of parasitic infection (*P* = 0.001) (Table 5).

### 5.6. Clinical Symptoms Associated with Infestation of Intestinal Parasitic Infections

In this study, parasitic infection was highest among symptomatic children with abdominal cramps (18; 58.06%) and constipation (2; 66.66%) than in asymptomatic children (26; 7.10%). There was significant difference between clinical symptoms and parasitic infection (*P* = 0.001) (Table 6).

### 5.7. Binary and Multiple Logistic Regression Results

Binary logistic regression analysis was performed to assess the independent effect of each factor. Multivariate logistic regression was performed for factors which are found to be associated with binary logistic regression analysis.

Nail-biting habit was found to be a strong significant predictor of intestinal parasitic infections (IPIs); nail biters are more likely to be infested with intestinal parasite than the nonbiters (AOR = 2.466, 95%CI = 0.830 − 7.333, *P* = 0.001). Source of water was also another strong determinant factor for IPIs; when compared with filtered water, direct tap water drinkers were more likely to be infested with IPIs (AOR = 2.218, 95%CI = 0.620 − 7.932, *P* = 0.001). Hand washing with water only after using the toilet was a significant risk factor for IPIs. Pupils who did not wash their hands with soap and water were more likely to be infested with IPIs than those who wash their hands only with water (AOR = 1.765, 95%CI = 0.139 − 4.216, *P* = 0.001). Hand washing with water alone before having contact with food was a significant risk factor of IPIs. Having information about awareness, especially about intestinal parasites, was strongly associated with IPIs. Parents who had no information had greater risk for IPIs (AOR = 8.226, 95%CI = 3.367 − 20.095, *P* = 0.001). Lack of wearing protective shoes at home and playgrounds was a significant risk factor for IPIs. Pupils who do not wear protective shoes were more likely infested by parasites than those who wear protective shoes (AOR = 33.212, 95%CI = 14.284 − 77.220, *P* = 0.001). Deworming status was the other strongly associated factor for IPIs. When compared with dewormed pupils, those without deworming had a greater risk of IPIs (AOR = 608.059, 95%CI = 131.049 − 2821.360, *P* = 0.001).

## 6. Discussion

Intestinal parasitic infections are among the most common infections worldwide [32]. It is the major cause of morbidity in school-aged children. Impairment of physical and mental development has also been identified as the worst effect of helminthic infection [33]. Distribution of parasitosis in humans depends upon various socioeconomic factors such as hygiene, availability of clean drinking water, and poverty [34].

The present study reported the prevalence of intestinal parasites to be 11.55% (46/400) among the private school-going pupils of Dharan. Similarly, the study by Shrestha et al. in 2019 reported similar prevalence of parasitosis (12.1%) among private school-going pupils which was in agreement to the present study [3]. However, this prevalence was not in agreement with the previous two studies from Dharan by Chongbang et al. in 2016 and by Gyawali et al. in 2010 [29, 30]. Similarly, the prevalence of intestinal parasitic infection in this study is lower than the reports from other areas of Nepal [18, 35]. Similarly, Tandukar et al. in 2013 reported slightly higher prevalence (16.7%) of parasitosis [22]. The present study reported the prevalence of *Ascaris lumbricoides*, *Enterobius vermicularis*, and *Trichuris trichiura* in significant numbers which was commonly reported even from studies by Bugssa et al. in 2015 and Regmi et al. in 2014 [18, 36]. The study by Shrestha et al. in 2012 reported higher protozoan infestation as compared to helminth infection [37]. However, in this study, helminthic infection was found higher than the protozoans, and overall prevalence was found to be comparatively low. The frequency of helminths in this study concords with the previous findings from Nepal [22]. Dharan is a comparatively developed as well as a clean and green city. The provision and effort for safe drinking water is made by chlorinating water in a reservoir. This effort has played a significant role in controlling the parasitosis in the population of Dharan. However, several other risk factors are associated behind the prevailing parasitosis.

This study reported a higher prevalence rate of parasitic infection in male (14.28%) than in female children (9%) with no statistically significant difference (*P* = 0.098) (Table 2). Sah et al. in 2013 reported intestinal parasitosis in males (12.4%) and in females (10.1%) [14]. Similarly, the study by Shrestha et al. in 2019 reported parasitosis in males (13.4%) and in females (10.5%) [3]. Both of these findings were quite similar to this present study. But according to two different studies by Tiwari et al. in 2013 and Rai et al. in 2017, the females were more susceptible to parasitosis than male populations which were not in agreement to this present study [4, 38]. However, Chandrashekhar et al. in 2005 reported that the prevalence was almost equal in both males and females [39]. The interrelationship between genders with parasitic infection may be affected by different socioeconomic aspects of places.

In this study, children from the age group of 6-8 years showed the highest prevalence of parasitic infection (13.55%) followed by the age group of 9-11 years (8.53%) with no statistical significance (*P* = 0.121) (Table 3). The study by Khanal et al. in 2011 showed that children from the age group of 6-8 years (21.4%) were more likely to be infected with parasites than children aged 9-12 years (18.6%) [21]. But, according to Dhital et al. in 2016, a greater number of infected children belonged to the age group of 9-12 years (32.19%) rather than from the age group of 6-9 years (24.42%) [5]. Carelessness towards personal hygiene and active involvement in outdoor physical activities could be the reason behind higher prevalence of parasitosis among the children 6-8 years of age. The level of awareness and knowledge is also comparatively more among children aged 9-11 years than among children aged 6-8 years [21]. The mass deworming program is not only sufficient in eradicating parasitosis in children. However, poor sanitary conditions, poor sanitary habits, and overcrowded environments of urban and semiurban areas have amplified the high infection and reinfection rates of parasitic infections in children [8].

Regarding ethnicity, the Dalit community children presented with highest rate of parasitosis followed by the Madhesi, Janajati, Chettri, and Brahmin communities. The variation in the prevalence percentage of parasitic infection by ethnic groups was statistically significant (*P* = 0.001). Higher prevalence of parasitosis among the Dalit as compared to the Aryans was also shown by two other studies done by Upama et al. in 2019 (71.4%) and Shrestha et al. in 2019 (36.2%) [3, 7] with significant statistical difference. In contrast, Janajati children were more prone to parasitic infection than Dalit students according to a study performed by Sah et al. in 2013, although it was not statistically significant [14]. Overall, risk factors can be responsible for increasing occurrence of parasitic disease among Janajati and Dalit communities.

The highest number of intestinal parasitic infections was seen in children drinking direct tap water (16.95%) and less among those drinking filtered or boiled water (4.16%). The association between the source of drinking water and prevalence of infection was statistically significant (*P* = 0.001) (Table 5). The outcome was in accordance with the two other studies conducted before by Dhital et al. in 2016 and Bertoncello et al. in 2017 which reported that the prevalence rate of parasitosis was significantly more in children drinking direct tap water [5, 8]. The contamination of drinking water sources due to open defecation, flood during rainy season, and disposal of human and animal wastes directly into rivers can be possible causes for higher cases of parasitic infections among untreated water consumers.

A good hygiene practice also plays a vital role in protection against different kinds of infections [40]. In this present study, children using soap and water for hand washing (5.48%) had lesser prevalence of parasitic infection than those washing hands only with water (52.77%). There was a significant relationship between hand wash hygiene and prevalence of parasitosis (*P* = 0.001) (Table 5). Studies by Das et al. in 2019 and Chongbang et al. in 2016 presented similar results where children washing hands without soap and water were more vulnerable to intestinal parasitic infestation [29, 41]. But in the study by Sah et al. in 2013, the frequency of parasitosis among students with good and poor hand wash hygiene was nearly equal [14]. Children who play outdoors and do not wash hands before meals and after using toilets have very high chances to come in contact with parasites [40].

In the present study, the prevalence of intestinal parasitic infestation was higher among pupils with finger nail-biting status. This result was in agreement with the studies conducted in Southern Brazil by Almeida et al. in 2017 and in Nepal by Sah et al. in 2013 [14, 42]. Lack of attention by the parents towards maintaining hand and nail sanitation of children is a possible reason behind the parasitic infestation. The nail-biting habit accounts for autoinfection and induces long-term parasitosis in children.

In this study, the prevalence of intestinal parasitic infestation was higher among pupils with no protective shoes during play. This finding was in agreement with the study conducted in Ethiopia by Gadisa and Jote in 2019 [43]. Higher prevalence among older children might be associated with their activities and behavior as children in this age group usually play and move around covering wider territory whereby the possibility of acquiring infections is increased [44]. In this study, the prevalence of intestinal parasitic infestation was higher among pupils with a thumb-sucking habit. This finding was in agreement with the study from Nepal by Sah et al. in 2013 [14].

The prevalence of intestinal parasitic infestation was higher among pupils having pet dogs/cats kept at home. Human infection with helminth parasites is an emerging health issue due to the sharing of the human environment with animals, either as pets or wild life [45]. Lack of sanitation and lack of deworming pet animals are associated factors behind zoonotic spillover of parasites from infected animals to children.

On the basis of a biannual deworming program, children who received anthelminthic drugs twice a year had very less chance of parasitic infection than those who did not participate in such program with a statistically significant difference (*P* = 0.001). A study by Gabrie et al. in 2014 conducted in Honduras also reported that the rate of parasitic infection was significantly more among school-going children who were either absent or received antiparasitic drugs once a year only [46]. In addition, Nanthavong et al. in 2017 concluded with a very similar result where the rate of prevalence of parasitosis among children was significantly decreased after the implementation of a biannual deworming program in Lao People's Democratic Republic (PDR) [47]. People who are aware of health and sanitation have administered their children with biannual anthelminthic drugs. However, the underprivileged population are negligent and never provide interest in participating in biannual deworming programs which induces in their children susceptibility to intestinal parasitosis.

Parasitic infection was comparatively more in pupils whose parents were unaware of knowledge regarding parasitosis, health, and sanitation. There was a statistically significant association between parent's awareness and parasitosis in children (*P* = 0.001). Similar results were reported from studies by Dhital et al. in 2016 and Al-Megrin in 2015 in Nepal where awareness in parents were linked with lesser occurrence of parasitic infection among children with statistical significance [5, 48]. However, a study performed in Ethiopia by Gebretsadik et al. in 2018 did not find any association between the awareness of parents and parasitic prevalence in children [49].

The children with clinical symptoms (58.82%) reported more cases of intestinal parasitic infection than without symptoms (7.10%) (Table 6). The clinical symptom was observed to be statistically significant with the prevalence of parasitosis (*P* = 0.001). This finding was in agreement with the studies by Yadav and Prakash in 2016 and Dahal et al. in 2018 which manifested significantly higher frequency of parasitic infection for symptomatic children [9, 40]. Abdominal cramps, discomfort, diarrhea, constipation, and nausea are the common symptoms of parasitic infestation [40].

Intestinal parasitic infection is a serious public health problem. In Nepal, a nationwide biannual deworming program by the administration of albendazole has contributed to eradicate parasitic infections. However, negligence in mass deworming program, lack of awareness, and poor sanitation and sanitary habits have always been factors behind parasitic transmission, infection, and reinfection. For the management of these infections, regular stool examination, deworming, and performance of good sanitary habits are necessary. Therefore, in this study the parents of infected pupils were informed about the lab report and were advised to seek further diagnosis and treatment from the nearby local hospitals.

## 8. Conclusion

This study concludes that intestinal parasites are copious among private school-going pupils of Dharan Submetropolitan City. The lack of hand-washing practice, nail-biting habits, source of drinking water, biannual deworming, thumb sucking, hand sanitation before having food and after using toilets, knowledge of parents on parasitosis, health and sanitation, keeping cats/dogs as pets, and status of wearing protective shoes were studied as contributors to the acquisition of intestinal parasitic infections among pupils. This situation highly suggests the immediate need for control measures, including the treatment of infected individuals, improvement of sanitation practices, and provision of safe drinking water. Furthermore, the need for regular screening of intestinal parasites and deworming among school children is required for effective management of these infections.

## Figures and Tables

**Table 1:** Prevalence of parasites.

Parasites	Prevalence (*n*, %)
Helminths	
*Ascaris lumbricoides*	22 (5.5%)
*Enterobius vermicularis*	6 (1.5%)
*Ancylostoma duodenale*	2 (0.5%)
*Trichuris trichiura*	13 (3.25%)
Protozoan	
*Entamoeba histolytica*	3 (0.75%)
Total	46 (11.5%)

**Table 2:** Gender-wise distribution of parasitic infection.

Gender	Total samples (*n*, %)	Prevalence of parasites (*n*, %)	Chi-square (*P* value)	Crude OR (CI)	Adjusted OR (95% CI)
Male	189 (47.25%)	27 (14.28%)	2.732 (*P* = 0.098)	0.594 (0.318-1.107)	7.048 (2.008-24.739)
Female	211 (52.75%)	19 (9%)			
Total	400	46			

**Table 3:** Age-wise distribution of parasitic infection.

Age group	Total samples (*n*, %)	Number of infected (*n*, %)	Chi-square (*P* value)	Crude OR (CI)	Adjusted OR (95% CI)
6-8	236 (59%)	32 (13.55%)	2.398	0.595	0.592 (0.491-0.715)
9-11	164 (41%)	14 (8.53%)	(*P* = 0.121)	(0.307-1.154)	
Total	400	46			

**Table 4:** Ethnicity-wise distribution of parasitic infection.

Ethnicity	Total samples (*n*, %)	Number of infected (*n*, %)	Chi-square (*P* value)	Crude OR (CI)	Adjusted OR (95% CI)
Brahmin	118 (29.5%)	4 (3.38%)		14.250 (4.157-48.852)	0.022 (0.005-0.087)
Chettri	92 (23%)	5 (5.43%)	53.33	8.700 (2.738-27.641)	0.017 (0.003-0.079)
Janajati	136 (34%)	16 (11.76%)	(*P* = 0.001)	3.750 (1.537-9.151)	0.052 (0.011-0.252)
Dalit	21 (5.25%)	10 (47.61%)		0.550 (0.179-1.688)	0.808 (0.190-3.431)
Madhesi	33 (8.25%)	11 (23.91%)			
Total	400	46			

**Table 5:** Risk factors associated with infestation of intestinal parasitic infections.

Characteristics	Total samples (*n*, %)	Number of infected (*n*, %)	Chi-square (*P* value)	Crude OR (CI)	Adjusted OR (95% CI)
Thumb-sucking habit					
Yes	72 (18%)	35 (48.61%)	118.81	0.037	0.447 (0.1436-1.467)
No	328 (82%)	11 (3.35%)	(*P* = 0.001)	(0.017-0.078)	
Parent's awareness					
Yes	246 (61.5%)	10 (4.06%)	34.70	7.20	8.226 (3.367-20.095)
No	154 (38.5%)	36 (23.37%)	(*P* = 0.001)	(3.454-15.010)	
Nail-biting habit					
Yes	184 (46%)	35 (19.02%)	18.94	4.378	2.466 (0.830-7.333)
No	216 (54%)	11 (5.09%)	(*P* = 0.001)	(2.153-8.90)	
Protective shoe-wearing habit					
Yes	343 (85.75)	14 (4.08%)	130.15	30.08	33.212 (14.284-77.220)
No	57 (14.25%)	32 (56.14%)	(*P* = 0.001)	(14.235-63.563)	
Have cats/dogs at home					
Yes	185 (46.25%)	34 (18.37%)	16.0	0.263	0.393 (0.146-1.059)
No	215 (53.75%)	12 (5.58%)	(*P* = 0.001)	(0.132-0.524)	
Biannual deworming					
Yes	347 (86.75%)	3 (0.86%)	291.06	493.067	608.059 (131.049-2821.360)
No	53 (13.25%)	43 (81.13%)	(*P* = 0.001)	(130.59-1861.668)	
Drinking water					
Direct tap water	230 (57.5%)	39 (16.95%)	15.832	0.210	2.218 (0.620-7.932)
Filtered/boiled water	170 (42.5%)	7 (4.11%)	(*P* = 0.001)	(0.092-0.483)	
Status of hand-wash hygiene					
With soap and water	328 (82%)	18 (5.48%)	64.718 (*P* = 0.001)	0.091 (0.047-0.179)	1.765 (0.139-4.216)
Without soap and water	72 (18%)	28 (38.88%)			

**Table 6:** Clinical symptoms associated with infestation of intestinal parasitic infections.

Clinical symptoms	Total samples (*n*, %)	Number of infected (*n*, %)	Chi-square (*P* value)	Crude OR (CI)	Adjusted OR (95% CI)
Abdominal cramps	31 (7.75%)	18 (58.06%)	94.983 (*P* = 0.001)	0.692 (0.057-8.470)	0.028 (0.010-0.705)
Constipation	3 (0.75%)	2 (66.66%)		0.038 (0.003-0.436)	
Asymptomatic	366 (91.5%)	26 (7.10%)			

## Data Availability

All data analyzed during this study are included in this article.

## Abbreviations

A.D.: Anno Domini
AOR: Adjusted odds ratio
CDC: Centre for Disease Control and Prevention
CI: Confidence interval
IPS: Intestinal parasitic infections
OR: Odds ratio
SPSS: Statistical Package for the Social Sciences
UNICEF: United Nation International Children's Emergency Fund
WHO: World Health Organization.
